# Pharmacokinetics and Bioavailability Study of Tubeimoside I in ICR Mice by UPLC-MS/MS

**DOI:** 10.1155/2018/9074893

**Published:** 2018-07-05

**Authors:** Lianguo Chen, Qinghua Weng, Feifei Li, Jinlai Liu, Xueliang Zhang, Yunfang Zhou

**Affiliations:** ^1^Wenzhou People's Hospital, The Third Clinical Institute Affiliated to Wenzhou Medical University, Wenzhou 325000, China; ^2^Laboratory of Clinical Pharmacy, The People's Hospital of Lishui, The Sixth Affiliated Hospital of Wenzhou Medical University, Lishui 323000, China

## Abstract

The aim of this study is to establish and validate a rapid, selective, and sensitive ultra-performance liquid chromatography-tandem mass spectrometry (UPLC-MS/MS) method to determine tubeimoside I (TBMS-I) in ICR (Institute of Cancer Research) mouse whole blood and its application in the pharmacokinetics and bioavailability study. The blood samples were precipitated by acetonitrile to extract the analytes. Chromatographic separation was performed on a UPLC BEH C18 column (2.1 mm × 50 mm, 1.7 *μ*m). The mobile phase consisted of water with 0.1% formic acid and methanol (1 : 1, v/v) at a flow rate of 0.4 mL/min. The total eluting time was 4 min. The TBMS-I and ardisiacrispin A (internal standard (IS)) were quantitatively detected by a tandem mass spectrometry equipped with an electrospray ionization (ESI) in a positive mode by multiple reaction monitoring (MRM). A validation of this method was in accordance with the US Food and Drug Administration (FDA) guidelines. The lower limit of quantification (LLOQ) of TBMS-I was 2 ng/mL, and the calibration curve was linearly ranged from 2 to 2000 ng/mL (*r*^2^ ≥ 0.995). The relative standard deviation (RSD) of interday precision and intraday precision was both lower than 15%, and the accuracy was between 91.7% and 108.0%. The average recovery was >66.9%, and the matrix effects were from 104.8% to 111.0%. In this assay, a fast, highly sensitive, and reproducible quantitative method was developed and validated in mouse blood for the first time. The absolute availability of TBMS-I in the mouse was only 1%, exhibiting a poor oral absorption.

## 1. Introduction

Tubeimoside I (TBMS-I), a triterpenoid saponin, is derived from the traditional Chinese bulb of *Bolbostemma paniculatum* (Maxim.). It was often used for the treatment of poisonous snake bite and inflammation [1]. In the past couple of years, additional attention was drawn to TBMS-I as it was reported to be a potential anticancer agent and appeared to be effective against several types of cancer, such as gliomas, breast cancer, colon cancer, and non-small cell lung cancer [2–5].

Pharmacokinetic studies are important in drug research and development which can provide systemic concentrations and exposure times of the drug for predicting a diverse range of efficacy- and toxicity-related events. To systematically examine the preclinical pharmacokinetic studies of TBMS-I in a reproducible and precise manner, a sensitive, fast, and validated analytical method for the determination of TBMS-I in biological fluids is imperative. However, the research on pharmacokinetics of TBMS-I lags behind compared to its pharmacological studies. Up to January of 2018, there was only one study report that published high-performance liquid chromatography coupled with tandem mass spectrometry (LC-MS/MS) for the determination of TBMS-I, which has been applied to the pharmacokinetic study in rats in 2007 [6]. But it had several drawbacks, such as long analysis time (more than 6 min) and low sensitivity (20 ng/mL), especially requiring a large volume of plasma (100 *μ*L), which make it unsuitable for the serial blood sampling in mice pharmacokinetic evaluation [7]. In the last couple of years, the UPLC technique has attracted more and more concern along with the development of analysis techniques [8]. Compared with LC-MS/MS, the UPLC-MS/MS method was faster, more sensitive, and with higher sample throughput [9, 10]. Meanwhile, its strong ability of isolating was more suitable for the analysis of the metabolism *in vivo* of the complex traditional Chinese medicine and complex compound [11, 12].

To the best of our knowledge, the profile of toxicity or pharmacokinetics of some drugs is alterable in different species [13–16], so it is not reasonable that these pharmacokinetic data are used directly in mice [17]. The mouse was chosen as the animal model to study the pharmacokinetics of TBMS-I in our study not only because of the reason mentioned above but also because the mouse is the most frequently used species for the preclinical efficacy [18, 19], toxicology [20], biodistribution [21], and pharmacokinetic [22] studies to evaluate a potential anticancer agent, particularly with a limiting drug supply or specialized animal models in the early new drug discovery stage [7, 23].

Thus, we established a rapid, sensitive, and selective UPLC-MS/MS method to quantitate the concentration of TBMS-I directly in the mouse utilizing low-volume whole blood after intravenous and oral administration in this study for the first time. A validation of this method was in accordance with the FDA guidelines. This method was successfully applied to the pharmacokinetics and bioavailability of TBMS-I in mice.

## 2. Materials and Methods

### 2.1. Experimental Materials

**Figure 1:**
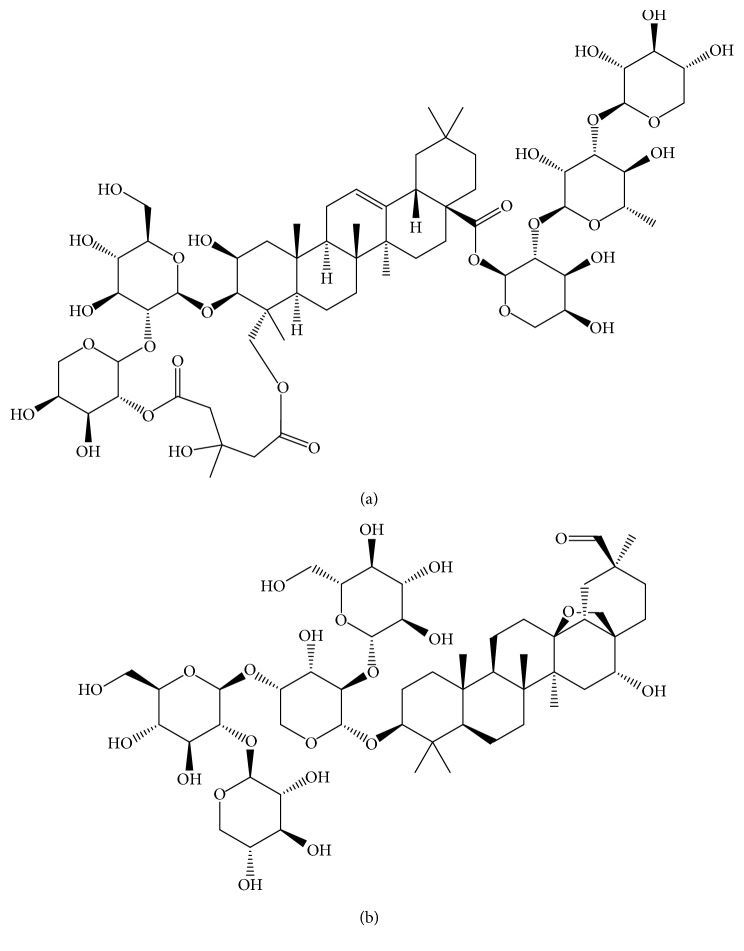
Chemical structures of tubeimoside I (a) and ardisiacrispin A (b).

TBMS-I (purity >98%; Figure 1(a)) and ardisiacrispin A (internal standard, purity >98%; Figure 1(b)) were purchased from Chengdu Mansite Biotechnology Co., Ltd. (Chengdu, China). HPLC-grade methanol and acetonitrile were bought from Merck (Darmstadt, Germany). HPLC-grade formic acid was supplied by Tedia (Ohio, USA). A Milli-Q system (Millipore, Bedford, USA) is used for generating ultrapure water. The ICR mice (male, weight 20–22 g, *n*=12) obtained were from the Laboratory Animal Center of Wenzhou Medical University (Wenzhou, China).

### 2.2. UPLC and Mass Spectrometric Conditions

The determination of analytes was carried out using the ACQUITY UPLC I-Class system equipped with a triple-quadrupole mass spectrometer (Waters Corp., Milford, MA, USA). MassLynx 4.1 software (Waters Corp.) was used to collect data and control the system.

TBMS-I and IS were separated on a UPLC BEH C18 column (2.1 mm × 50 mm, 1.7 *μ*m) with a stable temperature of 40°C. The mobile phases A and B were methanol and water with 0.1% formic acid, respectively. The details of gradient elution were as follows: the percentage of methanol was kept at 10% from 0 to 0.2 min and it reached 80% within 1.3 min; then, it was kept at the same percentage for 0.5 min and subsequently it turned back to 10% for another 0.5 min, and finally it was maintained at 10% for 2.5 min. The flow rate was set at 0.4 mL/min, and the total elution time was 4.0 min.

The mass spectrometer system for analysis was equipped with an electrospray source ionization (ESI) in a positive mode. The quantitative detection was performed in a multiple reaction monitoring mode at transitions *m*/*z* 1319.7 → 1187.6 for TBMS-I (collision voltage 12 V and cone voltage 30 V) and *m*/*z* 1083.5 → 407.1 for IS (collision voltage 72 V and cone voltage 100 V). The capillary voltage was 2.3 kV. High-purity nitrogen as curtain gas and drying gas was set at 50 L/h and 800 L/h, respectively. The temperature of the ion source and dissolvent was 150°C and 400°C, respectively.

### 2.3. Preparation of Stock Solutions, Quality Control (QC) Samples, and Calibration Standards (CS)

TBMS-I and IS were separately dissolved in methanol at a final concentration of 1.0 mg/mL as stock solutions. The working standard solutions were diluted from the stock solution using methanol. The standard working solution of IS was diluted with acetonitrile to the concentration of 50 ng/mL. All solutions were stored at 4°C prior to analysis.

CS samples were prepared by diluting blank mouse blood into corresponding standard working solutions. A series of concentrations of standard solutions were prepared with TBMS-I stock solutions and serially diluted by using methanol. The final concentrations of TBMS-I were from 2 to 2000 ng/mL, including 2, 5, 10, 20, 50, 100, 200, 500, 1000, and 2000 ng/mL.

Low-, mid-, and high-level QC samples of TBMS-I were similarly prepared at finial concentrations of 3, 190, and 1900 ng/mL, respectively. All the solutions were stored at −20°C until processed.

### 2.4. Sample Preparation

A 20 *μ*L aliquot of the mouse blood sample and 100 *μ*L of acetonitrile containing 50 ng/mL IS were added into 1.5 mL EP tubes [24]. After vortexing for 1 min, the specimens were centrifugated (13000 rpm) for 10 min at 4°C. Then, 80 *μ*L of the supernatant was collected; subsequently, a 2 *μ*L of the supernatant was injected into the UPLC-MS/MS system for analysis.

### 2.5. Method Validation

A validation of this method was in accordance with the FDA guidelines, including selectivity, linearity, precision, accuracy, recovery, matrix effect, and stability [25].

The chromatograms of blank mouse blood, blank blood spiked with TBMS-I and IS, and the real sample from mouse after dosing were used to estimate the selectivity of the UPLC-MS/MS method.

Calibration curves were generated by analyzing different concentrations of calibration samples on three consecutive days. The linear regressions of the peak area ratios (*y*) of each TBMS-I to the corresponding IS versus the nominal concentration (*x*) of TBMS-I were fitted over the range 2–2000 ng/mL. Linearity was evaluated covering the concentration range 2–2000 ng/mL.

The interday precision, intraday precision, and accuracy were estimated by determining three concentrations of quality control samples (*n*=6) on the same day and on three days in a row.

The recovery was calculated by comparison of the peak areas of TBMS-I and IS in the extracted low (3 ng/mL), middle (190 ng/mL), and high (1900 ng/mL) concentrations of QC samples with those of the extracted blank blood spiked with TBMS-I and IS at corresponding concentrations.

Matrix effects were tested by comparison of the peak areas of these new working solutions with those of the corresponding standard solutions diluted with methanol : 0.1% formic acid (1 : 1, v/v) at equivalent concentrations, and this peak area ratio is defined as the matrix effect.

The stability of TBMS-I was tested under four conditions: storage in an autosampler at 4°C, storage at room temperature for 2 hours, storage at −20°C for a month, and three complete freeze-thaw cycles (from −20°C to room temperature).

The stability of TBMS-I in mouse blood was obtained by comparing the areas of the newly configured QC samples with the corresponding three concentrations (3, 190, and 1900 ng/mL) of standard samples.

### 2.6. Pharmacokinetic Study

Twelve mice were randomly and equally divided into two groups (A and B). Mice in the group A were injected sublingually with 5 mg/kg TBMS-I, and mice in the group B were given TBMS-I orally at a final concentration of 20 mg/kg. The study protocol was approved by the Animal Care and Use Committee of Wenzhou Medical University. Mice were allowed to receive standard food and water ad libitum in a temperature-controlled room (25°C) with a 12-hour on and 12-hour off light cycle before the experiment.

Blood samples (20 *μ*L) were obtained from an individual mouse by tail vein bleeding in 1.5 mL tubes at 0 (prior to dosing), 0.0833, 0.5, 1, 1.5, 2, 3, 4, 8, 12, and 24 h after dosing. Six separate mice were used for sample collection and analysis at each time point. All the blood samples were directly stored at −20°C until analysis. DSA 2.0 pharmacokinetic software (China Pharmaceutical University, China) was used to calculate the main pharmacokinetic parameters, including the area under the time-concentration curve (AUC), half-life (*t*_1/2_), the maximum of blood concentration(*C*_max_), blood clearance rate (CL), apparent volume of distribution (*V*), and mean retention time (MRT). Bioavailability was calculated by absolute bioavailability = 100% × AUC_po_ · *D*_iv_/(AUC_iv_ · *D*_po_), where AUC_iv_ and AUC_po_ are the AUC of the drug from (0–∞) after intravenous and oral administration, and *D*_iv_ and *D*_po_ are the single dosage of TBMS-I for the intravenous and oral administration, respectively.

## 3. Results and Discussion

### 3.1. Method Optimization

**Figure 2:**
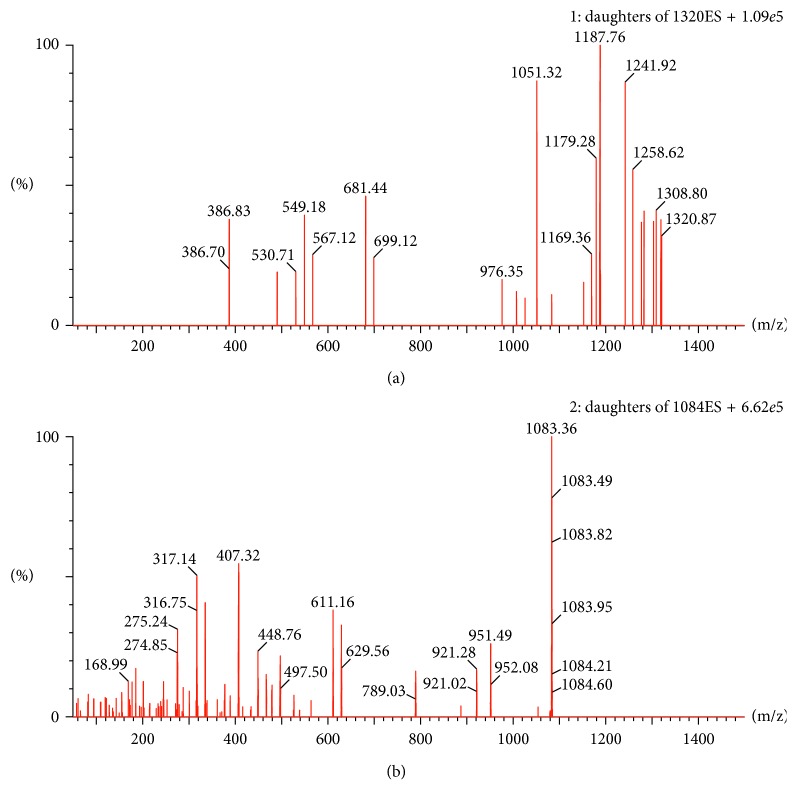
Mass spectrum of tubeimoside I (a) and ardisiacrispin A (b).

The mode of electronic source ionization (positive- or negative-ion mode) selection was often tested in a methodological study [26–30]. In this study, we chose the positive mode for the higher response achieved. According to the optimized results of mass spectrometric conditions, we can see that the daughter ions at *m*/*z* 1187.8 and *m*/*z* 407.3 were the strongest and the most stable among abundant fragment ions produced, respectively, by TBMS-I and IS, which was presented in Figure 2. Thus, we selected *m*/*z* 1319.7 → 1187.8 and *m*/*z* 1083.4 → 407.3 for TBMS-I and IS, respectively.

In order to wash out the endogenous compounds as much as possible and avoid endogenous interference, the mobile phase was optimized [31, 32]. Several mobile phases were investigated on the ACQUITY BEH C18 column to obtain a perfect separation and a more symmetrical peak shape [33], including acetonitrile and water with 0.1% formic acid, acetonitrile and 10 mmol/L ammonium acetate solution (0.1% formic acid), methanol and water (0.1% formic acid), and methanol and 10 mmol/L ammonium acetate solution (0.1% formic acid). Among this, the mobile phase containing the mixture of methanol and water (including 0.1% formic acid) was chosen in this study for the best mass spectrometry peak and retention time using gradient eluting.

Proteins and other potential interference would affect the analysis of the mass spectrometry system [34, 35]. Therefore, an effective and simple sample preparation was a key point for establishing the UPLC-MS/MS method of TBMS-I. Liquid-liquid extraction (LLE) has the advantages of the high extraction rate and low limit of quantification [36], but this method required a long time for evaporation of the extracting solvent and large sample volumes. The number of blood samples, which can be taken from a mouse (∼20 g), is limited. Thus, it is not easy to get enough plasma after centrifuging for liquid-liquid extraction at each point (10 total time points in 24 h) by tail vein transection bleeding. Taking these factors into consideration, only 20 *μ*L of blood samples was collected at different time points, and a one-step protein precipitation procedure for whole blood was chosen in our study following the example of the previous literature [37]. The small sample volume requirement further supports a serial blood sampling and enables entire pharmacokinetics from a single mouse which significantly reduces the numbers of mice used and inaccuracy of the pharmacokinetics because of individual differences [7, 23]. Our method provides a simple, direct, and high-throughput assay for measuring TBMS-I because of the simple sample processing. The following precipitating solvents and their mixtures in different combinations and ratios were tested: methanol, acetonitrile, and acetonitrile-methanol. The results indicated that acetonitrile was a good precipitating reagent for the best recoveries for the analytes. Considering that blood samples are more complex than plasma, 20 *μ*L of the blood sample was mixed with 5 volumes of acetonitrile, which can not only provide higher recoveries and less matrix effect but also provide a sufficient supernatant volume for multiple injections for analysis. The level of TBMS-I in the supernatant obtained from the blood after protein precipitation and centrifugation is high enough to be detected by UPLC-MS/MS because the LLOD is 0.7 ng/mL and LLOQ is 2 ng/mL for TBMS-I, which will contribute to the assay of lower concentration of TBMS-I at the last time point for sample collection.

Internal standard was also an important task for establishing this method [38–40]. Tubeimoside I and ardisiacrispin A had a similar structure, so the retention time and the way of ionization of them are similar. In addition, ardisiacrispin A was a good choice for IS in our study because of its robustness, stability, absence of matrix effects, and reproducible extraction.

### 3.2. Method Validation

#### 3.2.1. Selectivity

**Figure 3:**
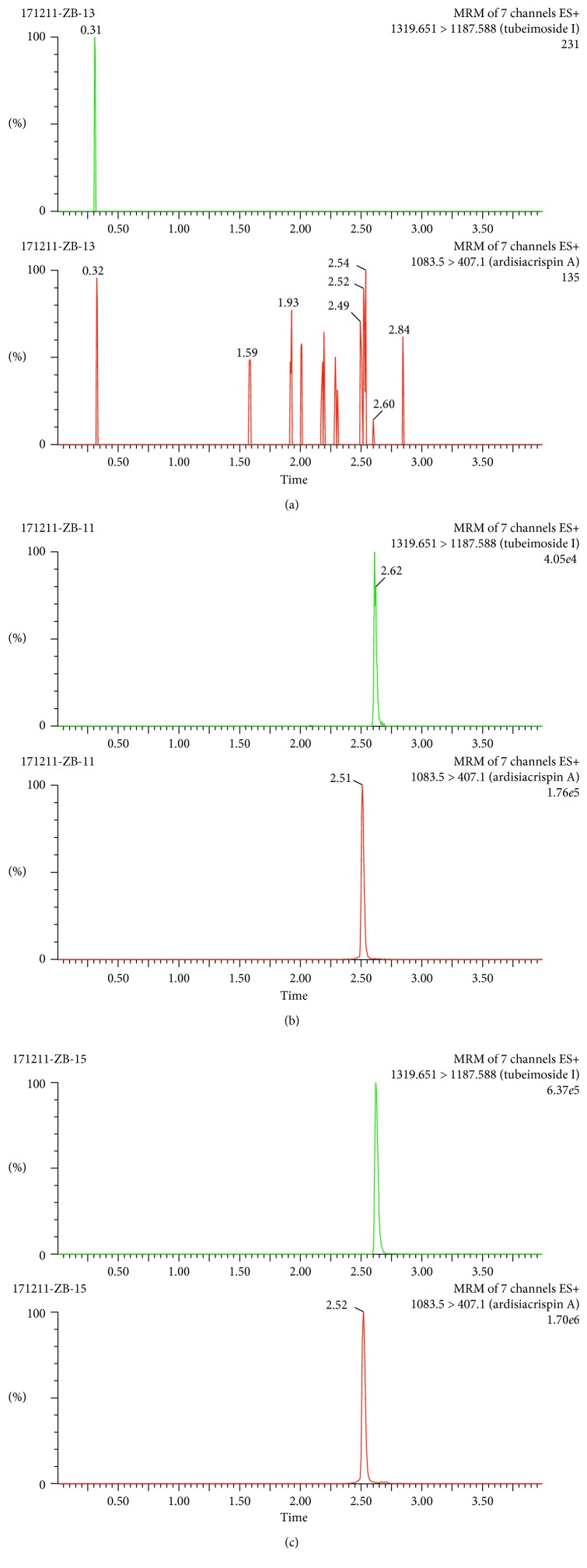
Spectrogram of the TBMS-I and IS: (a) a blank extract, (b) a blank extract with tubeimoside I and IS, and (c) the blood samples after administration spiked with IS.

Figure 3 presents the ion chromatogram of a blank extract, a blank extract with TBMS-I and IS, and a blood sample from the caudal vein spiked with IS. The peaks of TBMS-I and IS appeared at 2.62 and 2.52 min, respectively. No interfering peaks were found at or close by the retention times of TBMS-I and IS. The total runtime was 4.0 minutes.

#### 3.2.2. Linearity

The regression equation of the calibration curve of TBMS-I was *y* = 0.00027776*x* + 0.0000866688 (*y* represents the value of the peak area ratio of TBMS-I and IS and *x* represents the concentrations of TBMS-I in blood). The correlation coefficient *r*^2^ was 0.9976, which showed a good linearity. The LLOQ was 2 ng/mL with the signal-to-noise ratio (S/N ratio) of 10 for the determination of TBMS-I in mouse blood, and the lower limit of detection was 0.7 ng/mL with the S/N ratio of 3.

#### 3.2.3. Precision, Accuracy, Recovery, and Matrix Effect

Table 1 shows the results of the precision, accuracy, recovery, and matrix effect. The RSD of interday precision and intraday precision was no more than 14% and 15%, respectively. The accuracy was in the range of 91.7% to 108.0% at each QC level. All of the recoveries were above 66.9%, and matrix effects were between 104.8% and 111.0%. These data suggest that this method was satisfied with the pharmacokinetic study of TBMS-I.

#### 3.2.4. Stability

The blood samples under the different storage conditions mentioned above (*n*=3) were carried out the stability experiment (results shown in Table 2). In this study, the variations of each condition were within 14% and RSD was under 15%, which indicated a reliable stability behavior of TBMS-I under the different storage conditions.

### 3.3. Pharmacokinetic Studies

**Figure 4:**
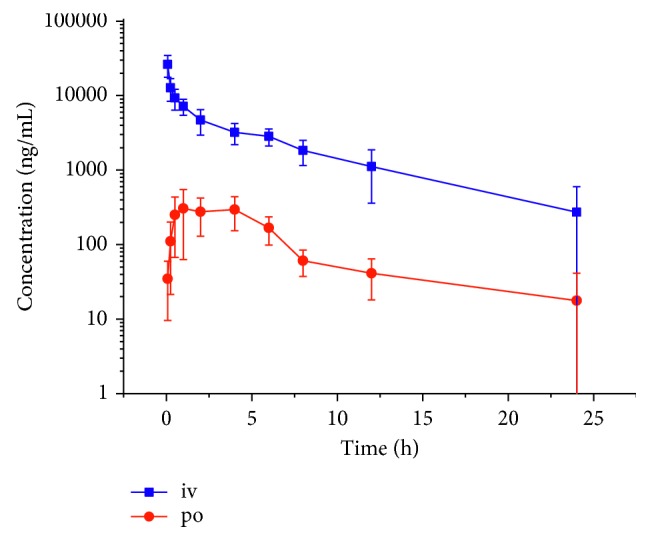
Mean blood concentration of TBMS-I after sublingual intravenous administration at the dose of 3 mg/kg and gavage of 15 mg/kg.

Time-concentration curve of TBMS-I after oral and intravenous administration is shown in Figure 4. The pharmacokinetic parameters were calculated according to the noncompartment model (results are presented in Table 3). The *t*_1/2*z*_ was 2.3 ± 0.5 h for oral administration and 6.8 ± 5.6 h for intravenous administration, respectively. The *T*_max_ was 1.8 ± 1.3 h after oral administration. The absolute availability was only 1.0%. These results in mice were similar to that of rats described by Liang et al. [6].

## 4. Conclusions

A novel UPLC-MS/MS method for the quantitative measurement of TBMS-I in mouse blood has been developed and validated. The application of this method for the determination of TBMS-I extracted from only 20 *μ*L of whole blood using a simple one-step protein precipitation procedure within 4 min was more sensitive, more convenient, and faster than traditional and commonly used analytical techniques. It is clear, in addition, that there are potentially large savings in the amount of compound and number of animals required for early pharmacokinetic studies. In the present study, the UPLC/MS/MS method has been successfully applied to the pharmacokinetic investigations of TBMS-I in mice after sublingual intravenous and intragastric administration. The oral bioavailability of tubeimoside I in mice is 1%, which indicates that tubeimoside I is not easily absorbed into blood circulatory system through the gastrointestinal tract.

## Figures and Tables

**Table 1:** Accuracy, precision, matrix effect, and recovery of the TBMS-I in mouse blood (*n*=6).

Concentration (ng/mL)	Precision (RSD %)		Accuracy (%)		Matrix effect (%)	Recovery (%)
	Intraday	Interday	Intraday	Interday		
3	13.4	14.5	105.8	108.0	105.2	78.4
190	11.2	10.6	94.4	91.7	104.8	68.6
1900	5.3	8.4	101.3	104.1	111.0	66.9

**Table 2:** The stability of TBMS-I under various storage conditions (*n*=3).

Concentration (ng/mL)	Autosampler (4°C, 12 h)		Ambient 2 h		−20°C 30 d		Freeze-thaw	
	Accuracy	RSD	Accuracy	RSD	Accuracy	RSD	Accuracy	RSD
3	96.0	3.8	107.5	5.1	92.6	14.2	113.5	8.8
190	106.2	6.7	108.0	4.8	109.9	9.4	111.0	11.2
1900	104.1	5.5	95.8	4.4	91.7	6.0	90.3	7.5

**Table 3:** The pharmacokinetic parameters of TBMS-I after oral and intravenous administration (*n*=6).

Parameters	Unit	iv (5 mg/kg)	po (20 mg/kg)
AUC (0–*t*)	ng/mL·h	51205.8 ± 13134.0	2023.9 ± 1145.5
AUC (0–∞)	ng/mL·h	59370.3 ± 21468.0	2051.8 ± 1106.5
MRT (0–*t*)	h	5.1 ± 1.8	4.6 ± 2.4
MRT (0–∞)	h	8.9 ± 7.0	4.9 ± 2.0
*t*_1/2*z*_	h	6.8 ± 5.6	2.3 ± 0.5
CL_*z*/*F*_	L/h/kg	0.1	16.3 ± 17.4
*T*_max_	H	—	1.8 ± 1.3
*V*_*z*/*F*_	L/kg	0.4	53.9 ± 56.9
*C*_max_	ng/mL	26192.5 ± 8491.9	429.6 ± 164.9
